# Comprehensive analysis of chloroplast codon usage patterns in the important medicinal genus *Panax*

**DOI:** 10.3389/fpls.2026.1875224

**Published:** 2026-07-22

**Authors:** Liang Wu, Xu Jia, Xingyue Fan, Yali Wang, Qiang Su, Ming Yang, Yaqin Hou

**Affiliations:** 1 Nanchong Key Laboratory of Individualized Drug Therapy, Department of Pharmacy, Beijing Anzhen Nanchong Hospital of Capital Medical University & Nanchong Central Hospital, The Second Clinical School of North Sichuan Medical College, Nanchong, Sichuan, China; 2 School of Pharmacy, Affiliated Hospital of North Sichuan Medical College, North Sichuan Medical College, Nanchong, Sichuan, China; 3 Department of Pharmacy, Xiamen Humanity Hospital, Xiamen, Fujian, China

**Keywords:** chloroplast genome, codon usage bias, synonymous codon usage, candidate optimal codons, codon adaptation index, plastomics, comparative genomics, Panax

## Abstract

**Introduction:**

The genus Panax comprises highly valued medicinal plants, yet codon usage patterns in its chloroplast genomes remain insufficiently characterized at the genus level. Here, we systematically investigated chloroplast codon usage bias (CUB) across nine Panax species and evaluated the potential factors shaping these patterns.

**Methods and results:**

Filtered coding sequences from the complete chloroplast genomes of nine Panax species were analyzed for nucleotide composition, relative synonymous codon usage, relative synonymous codon frequency, high-frequency codons, and candidate optimal codons. ENC-plot, PR2-plot, neutrality-plot, and correspondence analyses were performed to assess the contributions of compositional constraints, mutational bias, and possible selective effects. Codon-frequency patterns were also compared with those of four model organisms. Chloroplast genes exhibited moderate and highly conserved CUB across the genus, with a pronounced preference for A/T-ending codons. GC content at the third codon position ranged from 30.15% to 30.52%. Eleven candidate optimal codons were shared by all nine species, and all terminated in A or T. The combined ENC, PR2, neutrality, and correspondence analyses suggested that Panax chloroplast CUB was shaped by multiple factors rather than by GC-related mutational pressure alone. Nicotiana tabacum showed the greatest codon-usage similarity among the tested organisms.

**Discussion:**

The highly conserved A/T-ending codon preference provides a comparative reference for chloroplast genome evolution and codon optimization in Panax. However, the analytical approaches used here are indirect, and the candidate optimal codons should not be regarded as experimentally validated indicators of translational efficiency. Codon-usage similarity alone is also insufficient to predict heterologous expression efficiency. Nevertheless, these findings provide a useful resource for future chloroplast genetic-engineering studies.

## Introduction

The genus *Panax*, belonging to the Araliaceae family, comprises several perennial herbaceous plants such as *Panax ginseng* (*P. ginseng*), *P. quinquefolius*, and *P. notoginseng* that have been utilized as essential traditional medicines and modern functional foods (Bu et al., 2024; Zhao et al., 2025). These plants are rich in a diverse array of bioactive components, primarily including triterpenoid saponins, polysaccharides, and recently identified functional molecules like acetylcholine, which are widely distributed across various organs such as roots, leaves, flowers, and adapting seeds (Sun et al., 2023; Lee et al., 2026; Liu et al., 2026b; Luo et al., 2026). Modern pharmacological studies have demonstrated that these active constituents possess broad spectrum biological properties, including significant anti-inflammatory effects, neuroprotection against brain aging, and the critical maintenance of the blood brain and blood retinal barriers under oxidative stress (Liu et al., 2023; Peng et al., 2025; Fan et al., 2026; Zhang et al., 2026). Furthermore, *Panax* extracts and their specific compounds show immense clinical and therapeutic potential in preventing cardiovascular diseases, enhancing tumor chemosensitivity through apoptotic pathways, and significantly alleviating cancer related fatigue in survivors (Lan et al., 2026; Liu et al., 2026a; Rahmani et al., 2026).

Chloroplast genomes provide a valuable resource for phylogenetic inference and transgenic research in economically important plant species (Yu et al., 2021; Bulle et al., 2026). They are typically characterized by a stable, covalently closed circular DNA molecule with a highly conserved quadripartite structure, consisting of a large single-copy region and a small single-copy region separated by two identical inverted repeats (Daniell et al., 2016). Because chloroplasts are predominantly maternally inherited in angiosperms and exhibit limited recombination, chloroplast genomes retain relatively clear signals of evolutionary history and species divergence (Daniell et al., 2021; Fan et al., 2025). In addition to their phylogenetic utility, chloroplast genomes represent promising targets for genetic engineering. Their capacity for high-level transgene expression, together with maternal inheritance that restricts transgene transmission via pollen, makes them particularly suitable for applications requiring biological containment (Lee et al., 2021; Occhialini et al., 2022). To effectively exploit this system, a detailed understanding of codon usage patterns is required. Relative synonymous codon usage (RSCU) quantifies codon preference as the observed frequency of a synonymous codon relative to its expected frequency under equal usage (Chaney and Clark, 2015). Codon usage bias (CUB) arises from the combined effects of mutational pressure and natural selection. Mutation pressure reflects intrinsic biases in DNA replication and repair mechanisms, whereas natural selection optimizes translational efficiency and accuracy through correspondence with cellular tRNA abundance (Plotkin and Kudla, 2011; Parvathy et al., 2022; Hui et al., 2018; Iriarte et al., 2021).

Within the genus *Panax*, numerous chloroplast genomes have been sequenced, primarily to resolve phylogenetic relationships and develop molecular markers for species authentication (Zhao et al., 2014; Han et al., 2016; Nguyen et al., 2017; Jiang et al., 2018; Yang and Zhang, 2019; Nguyen et al., 2020). However, these studies have largely focused on single nucleotide polymorphisms, simple sequence repeats, and hypervariable intergenic regions for structural comparison and taxonomic discrimination, genome-wide patterns of codon usage evolution in *Panax* chloroplast genomes remain insufficiently explored. In particular, the identification of optimal codons, the characterization of CUB across species, and the quantitative assessment of the relative contributions of mutation and selection have not been systematically investigated at the genus level. Addressing these questions is essential for understanding the evolutionary dynamics of chloroplast genomes and for informing future chloroplast-based genetic engineering strategies in medicinal plants (Webster et al., 2017; Bock, 2022).

In this study, we systematically investigated codon usage patterns in the chloroplast genomes of nine *Panax* species. A comprehensive analysis was conducted on the filtered coding sequences (CDS) to evaluate base composition, RSCU, and to identify high-frequency and optimal codons. To disentangle the specific evolutionary forces shaping these patterns, we employed effective number of codons (ENC) plots, parity rule 2 (PR2) bias analysis, neutrality plots, and correspondence analysis (COA). Furthermore, we compared the codon usage frequencies of the *Panax* chloroplast genomes with four model organisms to evaluate their suitability as heterologous expression hosts. Ultimately, this study aims to elucidate the relative contributions of natural selection and mutational pressure to the evolution of *Panax* chloroplast genomes, while providing a robust theoretical basis and optimal codon references for future genetic engineering and efficient heterologous expression of *Panax* plastid genes.

## Materials and methods

### Genome data acquisition and preprocessing

The complete chloroplast genome sequences of nine *Panax* species were retrieved from the National Center for Biotechnology Information (NCBI) database (https://www.ncbi.nlm.nih.gov/nucleotide/). Annotated CDS and tRNA features were extracted and exported using Geneious Prime, and the exported CDS/tRNA tables were used as inputs for downstream table-based analysis scripts. To ensure the reliability of subsequent analyses, CDS sequences were retained only if they met the following criteria: sequence length was a multiple of three, sequence length was at least 300 bp, only standard nucleotides (A, T, G, and C) were present, sequences initiated with a canonical start codon (ATG) and terminated with a valid stop codon (TAA, TAG, or TGA), and no internal stop codons were present. The 300-bp threshold was used to reduce instability in codon-frequency estimates from very short CDSs; we recognize that this filter can exclude short plastid genes and therefore list all retained and excluded CDSs in **Supplementary Table 7**. For genes duplicated in inverted repeat regions, one representative copy was retained to avoid inflating codon counts by identical duplicated sequences. Gene names and homologous CDS sets were standardized across GenBank annotations before downstream analyses. The GC content at the first, second, and third codon positions (GC1, GC2, and GC3) was calculated for each species using the EMBOSS cusp tool based on all retained CDS sequences.

The annotated plastomes contained 36–37 tRNA features and 29–30 unique tRNA genes per species (**Supplementary Table 8**). These annotations were summarized to provide context for plastid translational machinery, but they were not used as expression-level evidence because tRNA gene copy number does not directly measure tRNA abundance. We also mapped the 11 shared candidate optimal codons to annotated plastid tRNA genes using tRNA anticodon labels and simple Watson-Crick/wobble pairing rules (**Supplementary Table 12**). This mapping was used as annotation-level compatibility context rather than as direct evidence of tRNA abundance or translational demand.

The 300-bp threshold was retained for the primary codon-usage analyses to reduce instability in codon-frequency estimates from very short CDSs. To evaluate whether this threshold affected the main conclusion, we performed a threshold-justification robustness analysis in which the length threshold was removed while all other CDS quality criteria were retained. For each species, we compared the primary 300-bp retained set with this no-length-threshold valid-CDS set and summarized the additional short CDSs, their codon-count contribution, weighted GC3, and A/T-ending codon proportions (**Supplementary Table 10**).

### Relative synonymous codon usage analysis

RSCU was calculated according to Sharp and Li (Sharp and Li, 1986) as $\mathrm{RSC}U_{ij}=\frac{X_{ij}}{\frac{1}{n_{i}}\sum_{j=1}^{n_{i}}X_{ij}}$, where X_ij_ represents the observed frequency of the *j*th codon encoding the *i*th amino acid and n_i_ denotes the number of synonymous codons for that amino acid. RSCU values greater than 1 indicate positive CUB, values less than 1 indicate negative bias, and a value equal to 1 indicates no bias. Codons with RSCU > 1.6 and RSCU< 0.6 were considered overrepresented and underrepresented, respectively (Xiao et al., 2024).

### Relative synonymous codon frequency and high-frequency codons

The relative frequency of synonymous codon usage (RFSC) was defined as the proportion of each codon among all synonymous codons encoding the same amino acid (Sharp and Li, 1986). A codon was classified as a high-frequency (HF) codon if its RFSC exceeded 0.60 or exceeded 0.5 × (1/n_i_) (Sheng et al., 2021).

### Identification of optimal codons

To identify candidate optimal codons, all CDS sequences were ranked according to their ENC values. The top one-third of genes with the lowest ENC values, representing genes with stronger codon bias rather than empirically measured high expression, were compared with the bottom one-third of genes with the highest ENC values. RSCU values were calculated separately for these two groups, and codons satisfying both RSCU > 1 and Delta RSCU (stronger-bias group minus weaker-bias group) > 0.08 were defined as candidate optimal codons (Shi et al., 2024). Because no transcriptomic, proteomic, or ribosome-profiling data were available for these Panax plastomes, these codons should be interpreted as codon-usage-inferred candidates rather than experimentally validated optimal codons. RSCU and ENC values were calculated using custom table-oriented scripts based on the CodonW 1.4.2/Sharp and Li calculation framework, and GC indices were calculated using EMBOSS cusp and script-based spreadsheet summaries. COA scores and axis-correlation summaries were also generated with the uploaded analysis scripts.

As an additional sensitivity analysis for candidate optimal codon inference, we calculated CAI values using *psbA* and *rbcL* as plastid reference genes and recalculated candidate codons by comparing the top and bottom thirds of genes ranked by this *psbA*/*rbcL*-based CAI (**Supplementary Table 11**). This analysis was used only to evaluate robustness to the reference-gene choice, not as independent expression validation.

### Analyses of factors influencing CUB

The ENC was calculated following Wright (Wright, 1990), with values ranging from 20 (maximum bias) to 61 (no bias). GC3s represents the GC content at synonymous third codon positions, excluding Met and Trp codons. An ENC–GC3s plot was constructed with GC3s as the x-axis and ENC as the y-axis, and the expected ENC under neutral mutational pressure was calculated as $ENC_{expected}=2+s+\frac{29}{s^{2}+{\left(1-s\right)}^{2}}$, where *s* denotes GC3s. Genes located below the expected curve suggest that CUB is influenced by factors other than mutational pressure, such as natural selection.

PR2 bias analysis was conducted by plotting G3/(G3 + C3) against A3/(A3 + T3), where A3, T3, G3, and C3 represent nucleotide frequencies at synonymous third codon positions, excluding Met and Trp codons. Deviation from the central point (0.5, 0.5) reflects unequal usage of complementary bases and indicates the combined effects of mutational bias and natural selection (Sueoka, 1995). Neutrality plot analysis was performed by regressing GC12, defined as the mean GC content at the first and second codon positions, against GC3. The slope of the regression line reflects the relative contribution of mutational pressure, whereas deviation from a slope of 1 indicates the influence of natural selection (Sueoka, 1995).

### Multivariate analysis of codon usage variation

COA was performed based on a 59-dimensional dataset of RSCU values (excluding Met, Trp, and stop codons) for all retained CDS sequences of each species. Pearson correlation coefficients were calculated between Axis 1, Axis 2, and four codon-related indices, including GC3s, codon adaptation index (CAI), ENC, and amino acid sequence length (L_aa_), to identify variables associated with codon usage variation (Romero et al., 2000). CAI was calculated using the stronger-bias gene group as a reference; because this reference was derived from ENC rather than independent expression data, CAI-based patterns were interpreted cautiously and were not treated as independent proof of translational selection.

### Comparative analysis of codon usage frequency

The codon usage frequency data of four model organisms were downloaded from the Kazusa Codon Usage Database (http://www.kazusa.or.jp/codon): A*rabidopsis thaliana* (*A. thaliana*, taxid: 3702), *Nicotiana tabacum* (*N. tabacum*, taxid: 4097), *Escherichia coli* (*E. coli*, taxid: 199310), and *Saccharomyces cerevisiae* (*S. cerevisiae*, taxid: 4932). These Kazusa records represent organism-level codon usage datasets rather than *Panax* plastid-specific expression systems. Therefore, the comparison was used only to assess codon-frequency similarity among the tested organisms, not to determine expression efficiency or host suitability by itself.

## Results

### Characteristics of codon base composition

**Figure 1:**
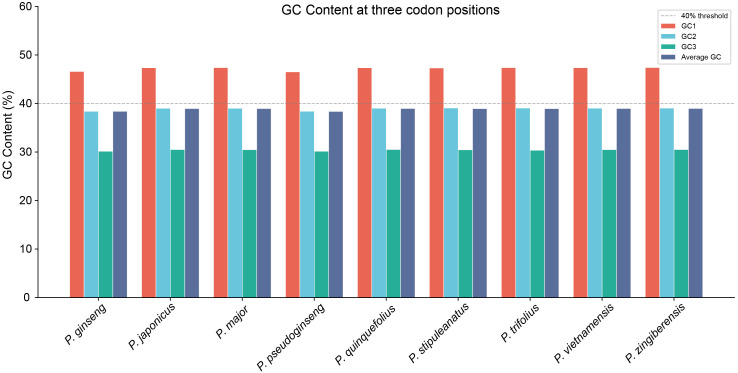
GC content at three synonymous codon positions in the chloroplast genomes of nine *Panax* species. The bar chart illustrates the percentage of GC content at the first (GC1), second (GC2), and third (GC3) codon positions, as well as the overall average GC content for each species. The horizontal dashed line indicates the 40% reference threshold.

After quality filtering and removal of redundant duplicated copies from inverted repeat regions, a total of 52 CDSs from the 79–80 annotated unique CDSs were retained for analysis in each of the nine Panax chloroplast genomes (**Table 1**; **Supplementary Table 7**). The retained set consisted of the same 52 orthologous CDSs in all nine species and was standardized across species to improve comparability; the exact retained and excluded CDS lists for each species are provided in **Supplementary Table 7**. Excluded genes were mainly short CDSs, noncanonical or partial annotations, or duplicated copies that did not meet the filtering criteria. The total amino acid lengths (L-aa) ranged from 19,226 (P. trifolius) to 20,714 (P. pseudoginseng), with a mean of 19,636 residues. The GC content at the three codon positions followed a consistent pattern across all nine Panax species, exhibiting a decreasing trend where GC1 > GC2 > GC3. Specifically, GC1 ranged from 46.52% to 47.40% (mean 47.19%), GC2 ranged from 38.39% to 39.07% (mean 38.89%), and GC3 ranged from 30.15% to 30.52% (mean 30.40%). These results indicate a consistent AT-ending tendency in synonymous codon usage across the retained Panax chloroplast CDSs (**Figure 1**) (Yu et al., 2020; Wang et al., 2022; Shen et al., 2024).

**Table 1:** Basic characteristics and nucleotide composition of the filtered chloroplast coding sequences (CDS) in nine *Panax* species.

Species	CDSs number (after processing)	L-aa	GC1 (%)	GC2 (%)	GC3 (%)	Average GC at three locations (%)	Accession no
*P. ginseng*	52	20,691	46.6	38.39	30.16	38.38	OL543607
*P. japonicus*	52	19,355	47.37	39.01	30.5	38.96	NC_028703
*P. major*	52	19,395	47.39	39.01	30.48	38.96	NC_053713
*P. pseudoginseng*	52	20,714	46.52	38.4	30.15	38.36	MW145449
*P. quinquefolius*	52	19,381	47.37	39.02	30.52	38.97	NC_027456
*P. stipuleanatus*	52	19,290	47.32	39.07	30.45	38.95	NC_030598
*P. trifolius*	52	19,226	47.39	39.06	30.37	38.94	NC_037994
*P. vietnamensis*	52	19,325	47.38	39.03	30.5	38.97	MF377623
*P. zingiberensis*	52	19,343	47.4	39.04	30.51	38.99	NC_043954

The retained CDS list was also used to verify that stop codons, Met, and Trp were excluded consistently from synonymous codon-based analyses where required, including RSCU, ENC-GC3s, PR2, neutrality, and COA calculations. The threshold-justification robustness analysis supported retaining the 300-bp threshold for the primary analysis. When the length threshold was removed, 66–78 valid CDSs per species passed the remaining quality criteria, adding 23–26 short but otherwise valid CDSs relative to the 52-CDS primary set (**Supplementary Table 10**). These additional genes were mainly short photosystem, ribosomal protein, ATP synthase, and cytochrome b6/f subunit genes, but they contributed only 6.34%-7.26% of the total codons in the no-length-threshold robustness sets. The A/T-ending tendency was unchanged: A/T-ending codons accounted for 69.41%-69.85% in the robustness sets compared with 66.90%-69.84% in the primary 300-bp sets, and weighted GC3 changed by -0.0003 to -0.0283. Thus, the 300-bp threshold improves stability and comparability while not changing the qualitative conclusion that *Panax* chloroplast CDSs show an A/T-ending codon preference.

### RSCU analysis and optimal codons

**Figure 2:**
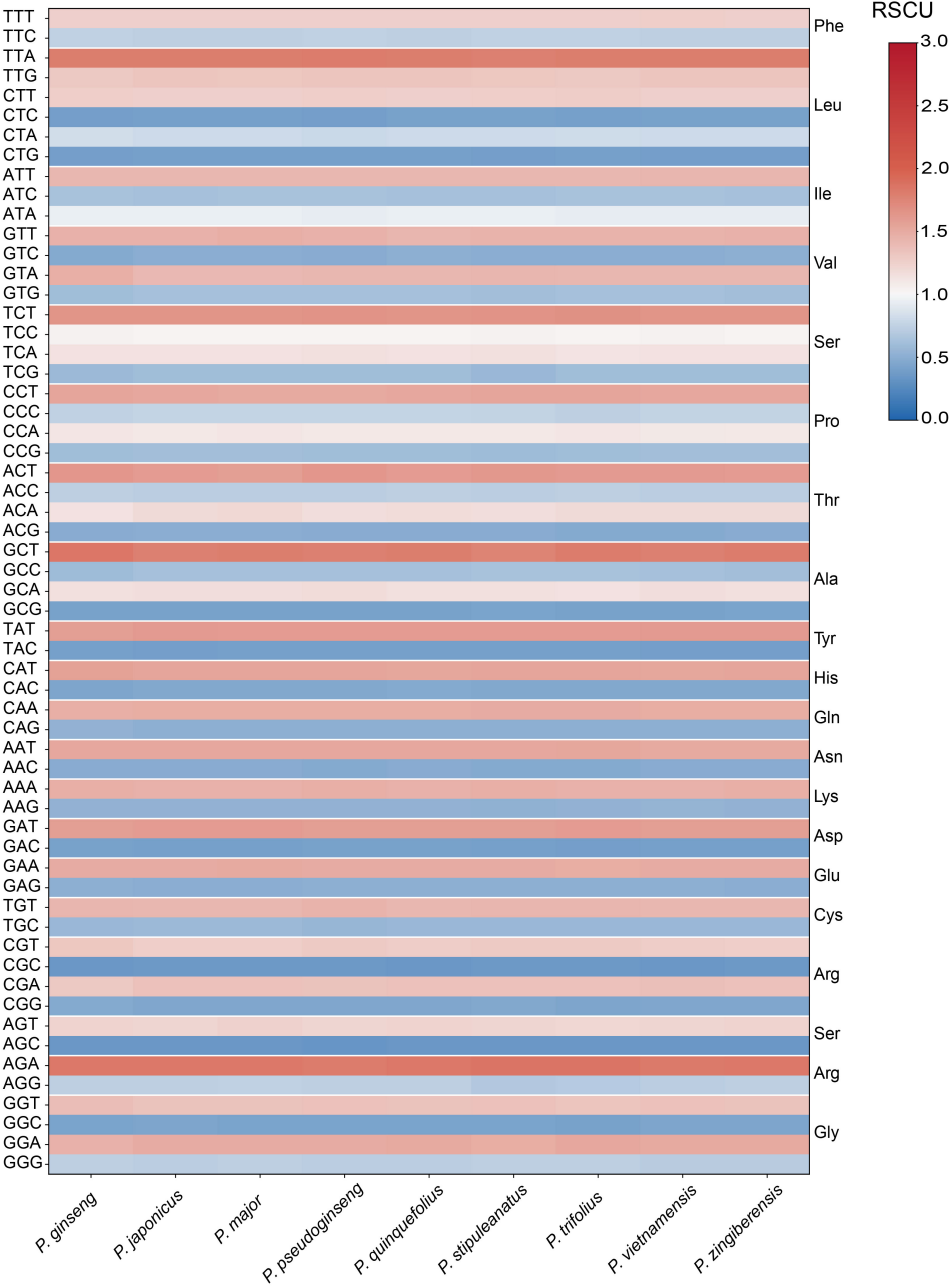
Heatmap of RSCU values in the chloroplast genomes of nine *Panax* species. The color gradient represents the RSCU values for all synonymous codons, with red indicating higher values (preferred usage) and blue indicating lower values (less frequent usage).

To further characterize synonymous codon usage preferences, RSCU values were calculated for all CDS of each species (**Figure 2**; **Supplementary Table 1**). The number of preferred codons (RSCU > 1) was 29 across all nine species, of which 28 ended in A or T, accounting for approximately 96.6% of all preferred codons. This finding is consistent with the A/T-ending bias observed in the base composition analysis. The codon with the highest mean RSCU across all species was TTA (Leu, RSCU ≈ 1.89), followed by GCT (Ala, 1.79), TCT (Ser, 1.71), AGA (Arg, 1.69), and ACT (Thr, 1.64) (**Supplementary Table 1**). Conversely, the lowest RSCU values were observed for AGC (Ser, 0.34), CGC (Arg, 0.35), CTC (Leu, 0.38), CTG (Leu, 0.38), TAC (Tyr, 0.39), and GGC (Gly, 0.44), all of which end in G or C. Five codons (TTA, GCT, TCT, AGA, and ACT) were overrepresented (RSCU > 1.6) in all nine species, while the most underrepresented codons (RSCU< 0.5) were predominantly G/C-ending. High-frequency (HF) codons numbered 44–46 per species, and a core set of 44 HF codons was shared across all nine *Panax* species (**Supplementary Table 2**). The vast majority of these preferred codons ended in A or T (with the sole G/C-ending exception of TTG, RSCU ≈ 1.28), further confirming the strong A/T-ending preference of *Panax* chloroplast genes.

**Figure 3:**
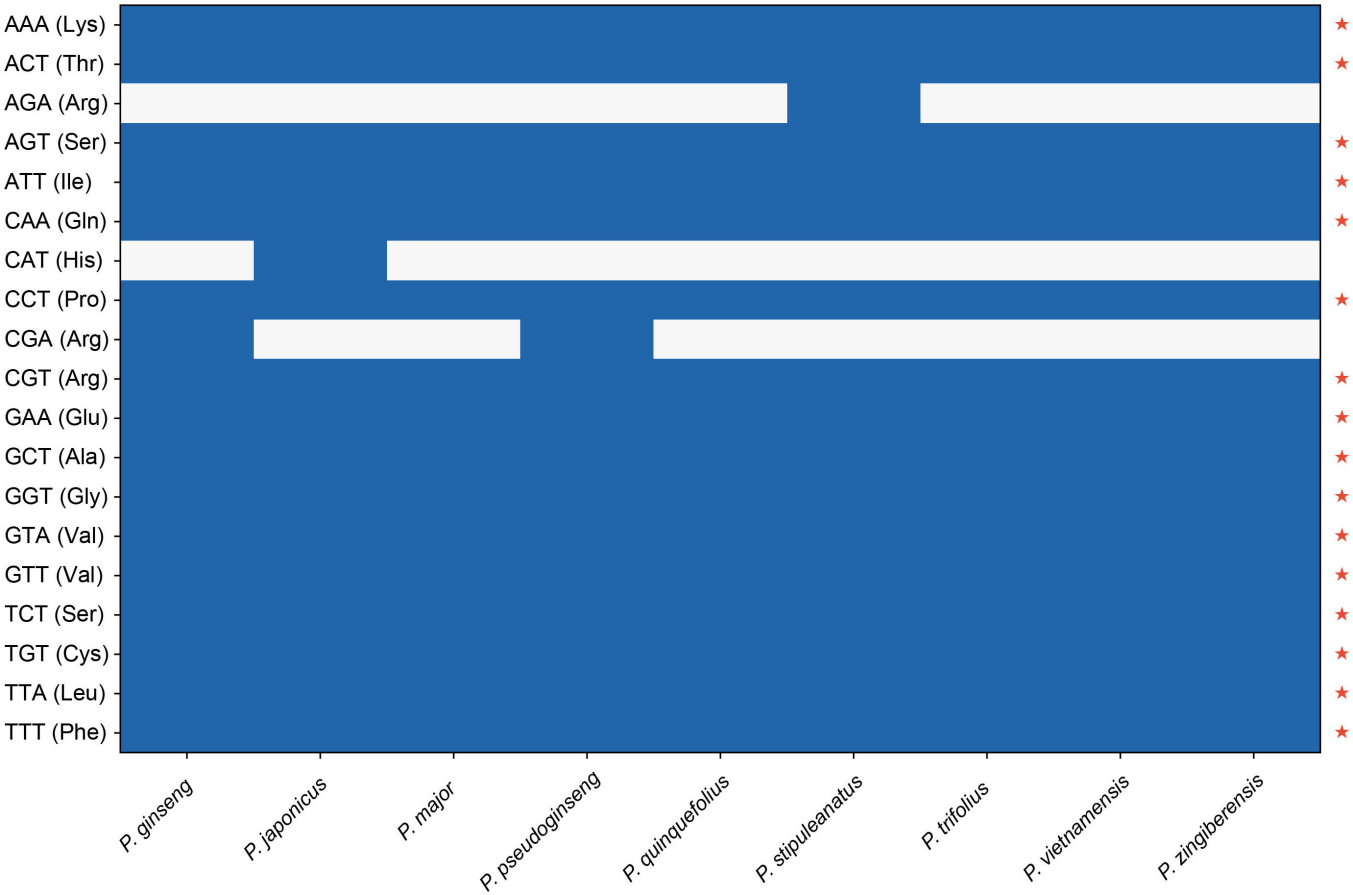
Distribution of candidate optimal codons across the chloroplast genomes of nine *Panax* species. Blue cells indicate that a specific codon was identified as a candidate optimal codon for the corresponding species. Red stars on the right highlight the 11 candidate optimal codons shared by all analyzed species.

Candidate optimal codons were identified based on RSCU values and codon-frequency criteria (**Figure 3**; **Supplementary Table 3**). The number of candidate optimal codons per species ranged from 14 (*P. ginseng*) to 17 (*P. vietnamensis* and *P. zingiberensis*). Across all nine species, 11 codons were shared: ACT (Thr), ATT (Ile), CAA (Gln), CCT (Pro), CGT (Arg), GAA (Glu), GGT (Gly), GTT (Val), TCT (Ser), TGT (Cys), and TTA (Leu). All 11 shared candidate optimal codons, as well as all 18 unique candidate optimal codons identified across the genus, were A/T-ending. Because the reference gene groups were inferred from ENC rather than measured expression, these codons are best regarded as candidate optimal codons inferred from codon-usage patterns. In the psbA/rbcL-based CAI sensitivity analysis, 15–19 candidate codons were identified per species, and 13–15 of these overlapped with the ENC-derived candidate codons in *each* species (**Supplementary Table 11**). Fourteen psbA/rbcL-reference candidate codons were shared across all species, of which nine overlapped with the 11 shared ENC-derived candidate codons. This substantial but incomplete overlap indicates that the candidate set is partly robust but remains reference-dependent.

### ENC-plot analysis

**Figure 4:**
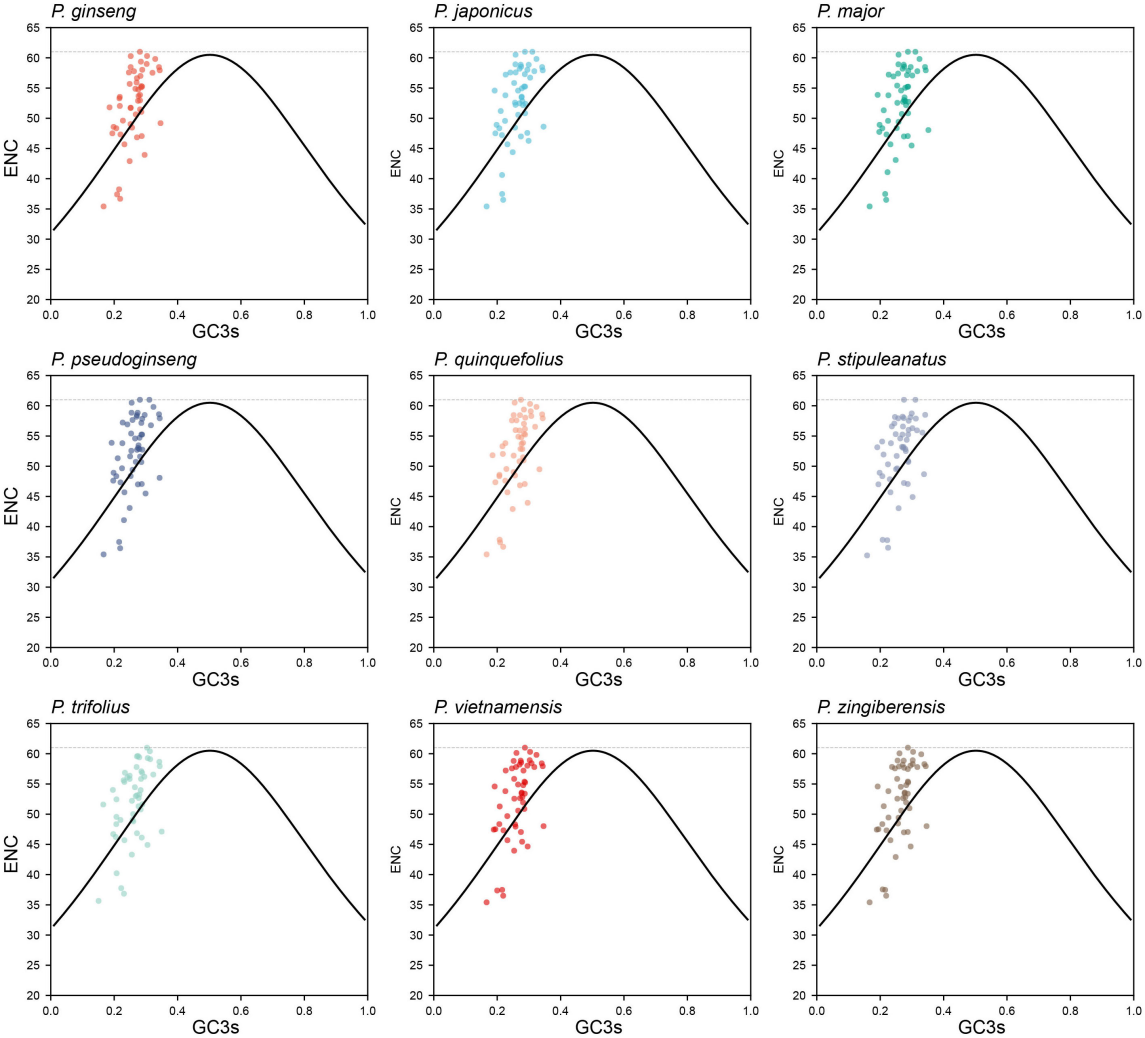
ENC-plot analysis of the chloroplast genes across nine *Panax* species. The solid black curve represents the expected ENC under pure mutational pressure. Each dot represents an individual protein-coding sequence plotted according to its actual ENC and GC3s values.

The ENC is a widely used index for quantifying the degree of CUB, with values ranging from 20 (extreme bias) to 61 (no bias). In the present study, the ENC values for individual genes ranged from 35.24 to 61.00 across all nine species (**Supplementary Table 4**; **Figure 4**). Mean ENC values per species were highly similar, ranging from 52.08 (*P. trifolius*) to 52.55 (*P. japonicus*), indicating a moderate but consistent degree of codon usage bias throughout the genus. Genes with the strongest codon bias (lowest ENC) included *rpl16* (mean ENC = 35.42), *ndhG* (36.57), and *petD* (37.52), while genes with the weakest bias (highest ENC) included *ycf3* (60.85), *atpE* (60.69), and *ycf4* (59.68). The mean GC3s values were uniformly low across all species (0.2611–0.2639), consistent with the AT-biased composition observed at the third codon position.

In the ENC-GC3s plots, the distribution of genes relative to the expected curve under pure mutational pressure was broadly consistent across all nine species (**Figure 4**; **Supplementary Table 9**). Using an on/near-curve tolerance of +/-0.5 ENC units, 103 of 468 genes (22.01%) were below the expected curve, 15 genes (3.21%) were on or near the curve, and 350 genes (74.79%) were above the curve. At the species level, 11–12 genes (21.15%-23.08%) were below the expected curve, 0–3 genes (0%-5.77%) were on or near the curve, and 38–40 genes (73.08%-76.92%) were above the curve. Therefore, the ENC plot does not support the previous statement that most genes fall below the expected curve. Instead, it indicates that many retained *Panax* chloroplast genes have ENC values equal to or higher than those expected from GC3s alone, whereas a smaller subset shows stronger codon bias than expected under the GC3s-based neutral model. We therefore interpret the ENC plot as an indirect descriptive analysis rather than as evidence for strong natural selection.

### PR2-plot analysis

**Figure 5:**
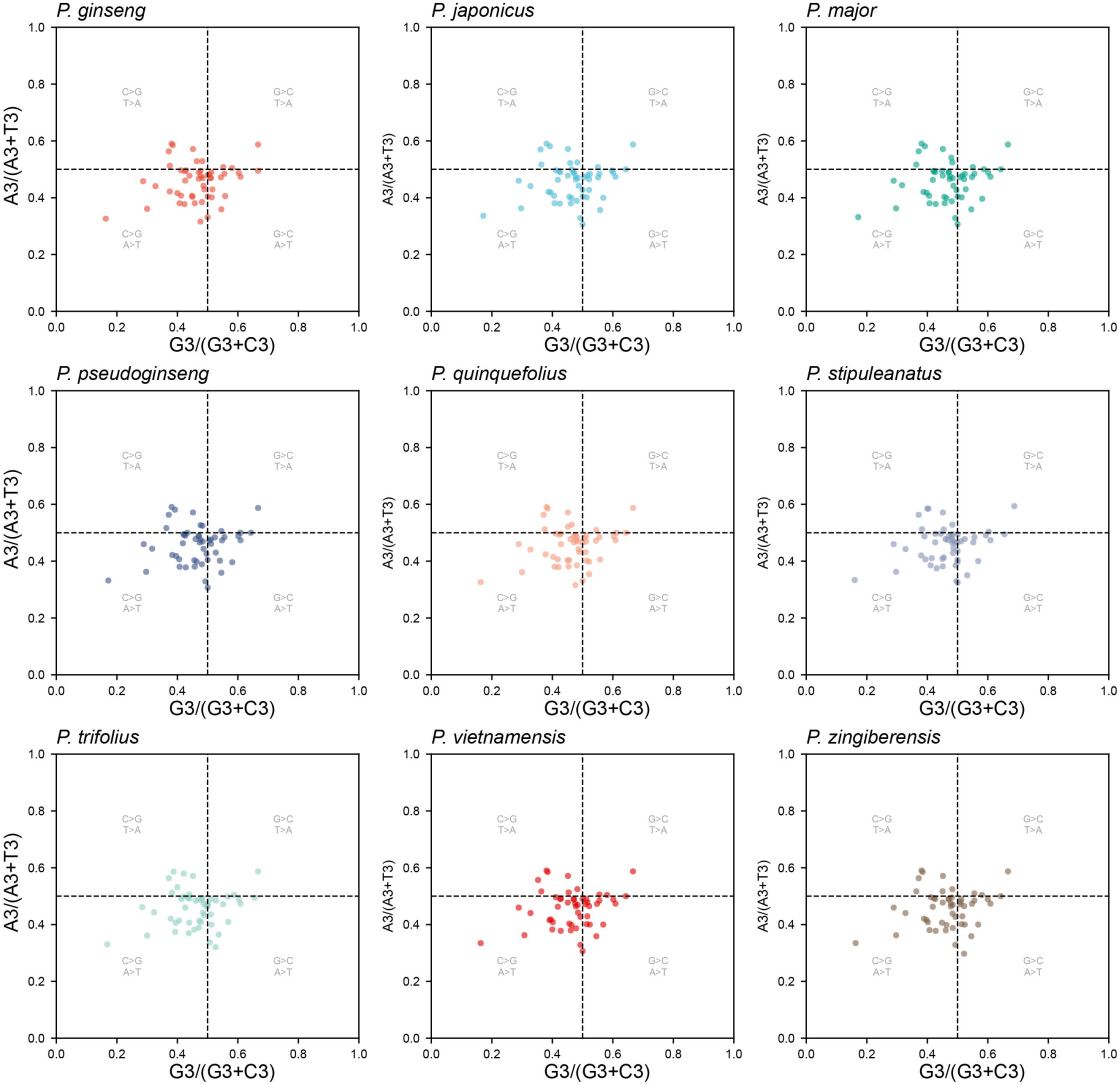
PR2 bias plot analysis of the chloroplast genes across nine *Panax* species. The plots illustrate the relationship between G3/(G3+C3) on the horizontal axis and A3/(A3+T3) on the vertical axis. The intersection of the dashed lines at (0.5, 0.5) indicates the theoretical position where complementary bases are used equally under pure mutational pressure.

PR2-plot analysis was performed to assess the relative usage of complementary bases (A vs. T and G vs. C) at the synonymous third codon position, providing insight into the relative contributions of mutational pressure and natural selection to CUB (**Figure 5**). Under the neutral model of pure mutational pressure, the frequencies of A and T, and of G and C, at the third codon position should be equal, placing all genes at the center of the plot (0.5, 0.5). Deviation from this center indicates the influence of natural selection or strand-specific mutational biases.

In all nine *Panax* species, the majority of genes were distributed in the lower-right quadrant of the PR2 plot, characterized by A3/(A3+T3)< 0.5 and G3/(G3+C3) > 0.5. This distribution indicates that T is used more frequently than A, and G is used more frequently than C, at the synonymous third codon position across the *Panax* chloroplast genome. These asymmetries are inconsistent with a simple expectation of equal complementary-base usage, but they cannot distinguish natural selection from strand-specific mutational bias or other compositional constraints on their own (Hui et al., 2018; Iriarte et al., 2021). We therefore interpret the PR2 results as evidence that mutational pressure alone is insufficient, while avoiding a definitive assignment of the deviation to natural selection.

### Neutrality plot analysis

**Figure 6:**
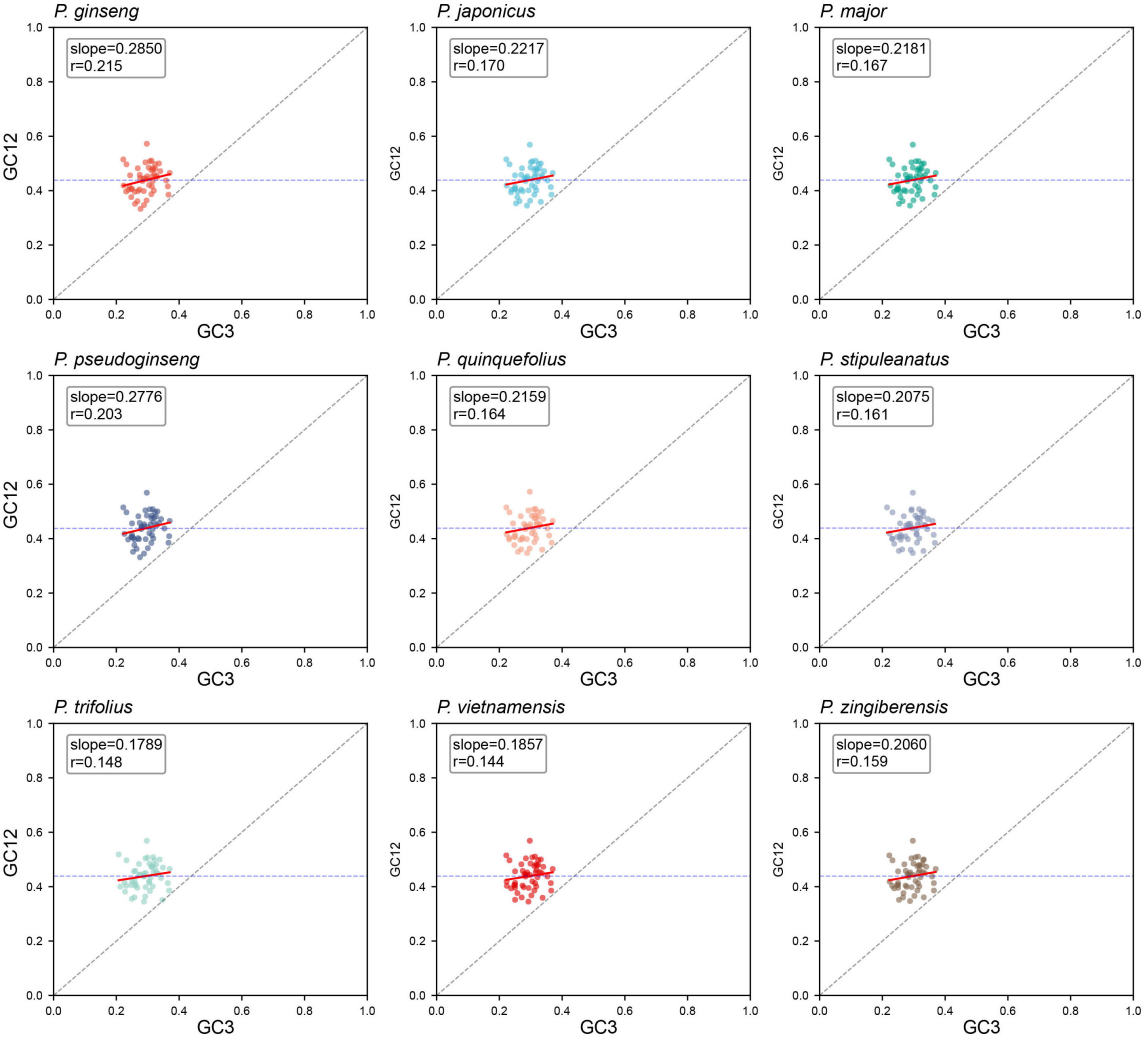
Neutrality plot analysis of the chloroplast genes across nine *Panax* species. The scatter plots illustrate the relationship between GC3 (x-axis) and GC12 (the average GC content at the first and second codon positions, y-axis) for individual genes. The solid red line represents the linear regression of the data, while the diagonal dashed line indicates the expected relationship under pure mutational pressure (slope = 1).

To further quantify the relative contributions of mutational pressure and natural selection to CUB, neutrality plot analysis was performed by regressing GC12 (the average GC content at the first and second codon positions) against GC3 (the GC content at the third codon position) for all CDS of each species (**Figure 6**). Under a model of pure mutational pressure, GC12 and GC3 should change in concert, yielding a regression slope of 1. Conversely, if natural selection acts predominantly on codon usage, GC12 and GC3 should be largely independent, yielding a slope approaching 0 (Sueoka, 1999).

The regression slopes across the nine *Panax* species ranged from 0.1789 (*P. trifolius*) to 0.2850 (*P. ginseng*), with a mean of 0.2218 (**Supplementary Table 5**). The corresponding slope-based estimates suggest a limited contribution of GC3-driven mutational pressure under the neutrality-plot framework. However, the correlation coefficients between GC12 and GC3 were weak (r = 0.1437-0.2147), and none reached statistical significance (p = 0.126-0.310 for all species). Therefore, the slope-derived percentages should be treated as approximate descriptive indicators rather than exact quantitative estimates of natural selection. These results support the conclusion that GC12 and GC3 are largely decoupled in the retained *Panax* chloroplast CDSs, but they do not provide definitive evidence for a precise selection-versus-mutation partition.

### Correspondence analysis

**Figure 7:**
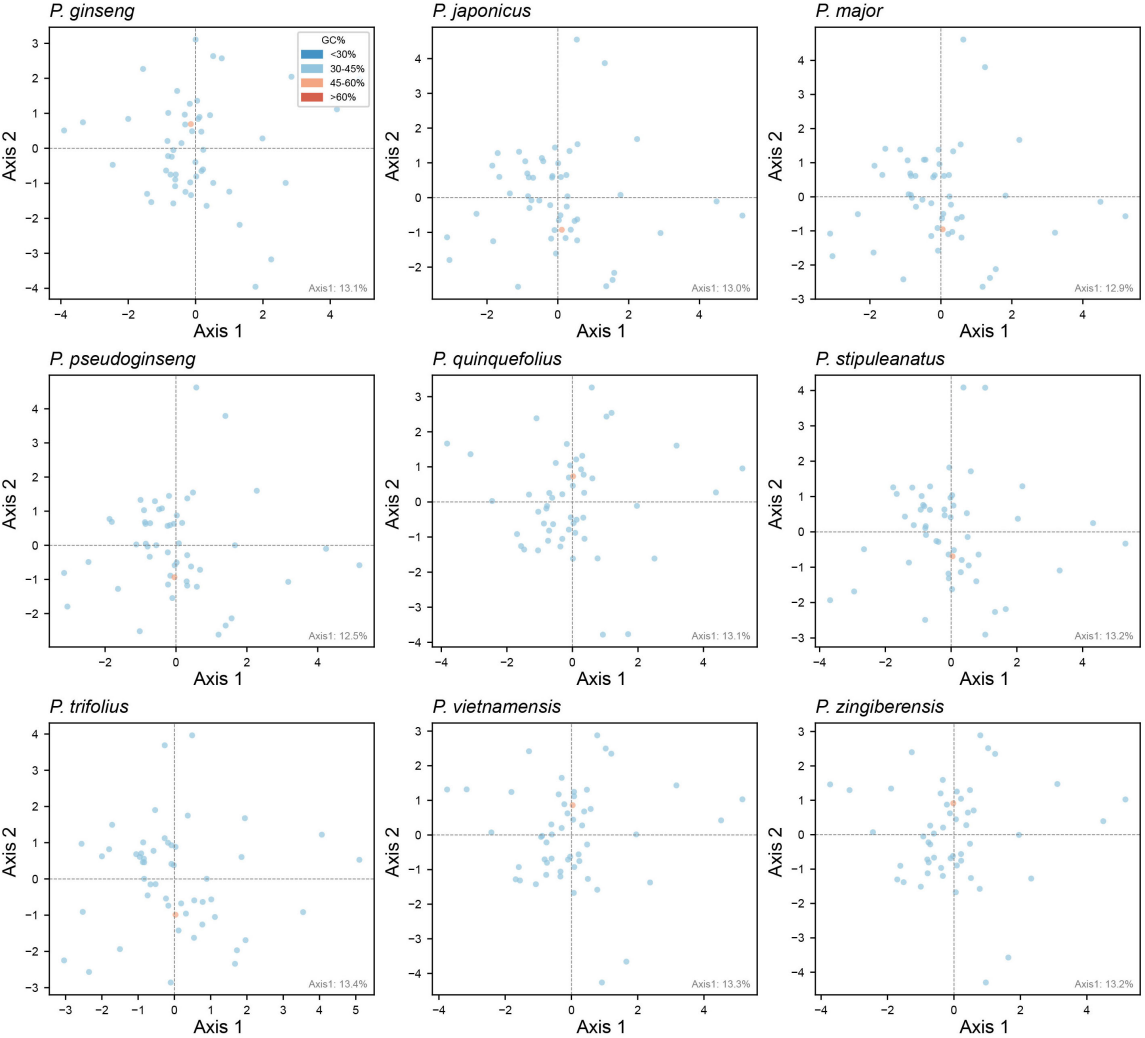
COA of synonymous codon usage in the chloroplast genomes of nine *Panax* species. The scatter plots display the distribution of individual genes along the first two principal axes of variation (Axis 1 and Axis 2). Each point represents a single coding sequence, color-coded based on its overall GC content.

COA was performed on the 59-dimensional RSCU vectors of all retained CDSs for each species to identify major axes of codon usage variation (**Figure 7**). Across the nine species, Axis 1 alone accounted for only 9.87%-10.63% of the total variation, while Axis 2 accounted for an additional 8.41%-8.70% of the variation. Together, the first two axes explained 18.28%-19.33% of total variation, leaving approximately 80.67%-81.72% distributed across higher axes. This modest explained variance indicates that codon usage variation is distributed across multiple axes rather than dominated by a single factor. The tight clustering of genes and the similar axis variance proportions across species are most consistent with a conserved codon usage architecture within *Panax*, but Axis 1 should not be overinterpreted as a dominant mechanistic driver.

**Figure 8:**
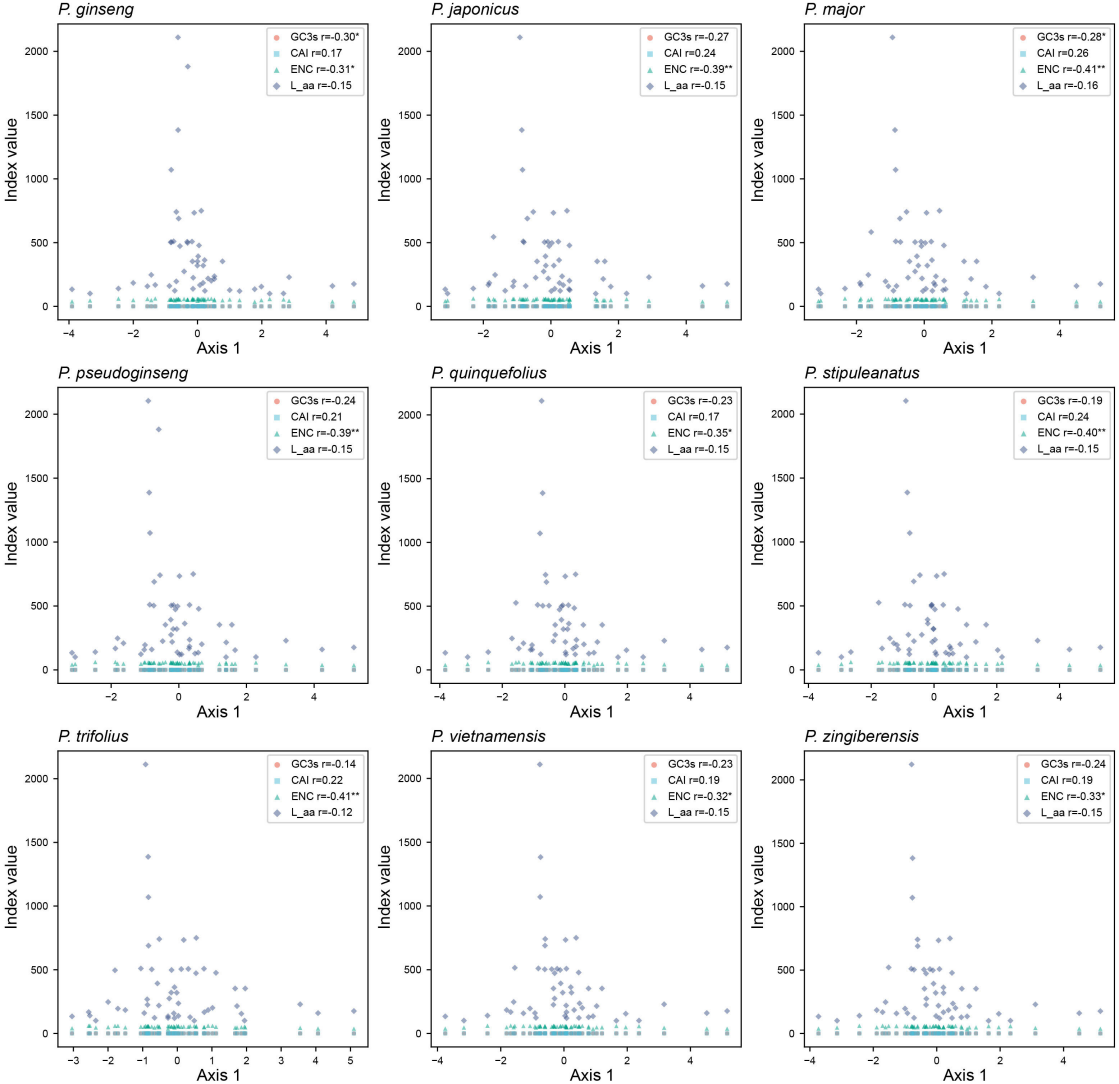
Correlation analysis between the first principal axis (Axis 1) and codon usage indices across nine *Panax* species. The plots illustrate the relationship of Axis 1 with four key variables: GC3s, CAI, ENC, and amino acid sequence length (L_aa). The Pearson correlation coefficients (r) are detailed in the legends of each panel, with asterisks denoting statistical significance.

To identify variables associated with the first two COA axes, Pearson correlation coefficients between Axis 1, Axis 2, and four codon usage indices (GC3s, CAI, ENC, and L_aa) were calculated for each species (**Figure 8**). ENC showed a significant negative correlation with Axis 1, and GC3s showed a significant positive correlation with Axis 1, indicating that codon bias and base composition are associated with the primary axis of variation. CAI was also correlated with Axis 1; however, because the CAI reference was derived from the ENC-defined stronger-bias gene group, this correlation is not independent evidence for gene expression level or translational selection. L_aa showed no consistent association with Axis 1. We therefore interpret the COA correlations as associations with codon bias and compositional features rather than definitive proof of translational selection.

### Comparative analysis of codon usage frequency

The codon usage frequencies of the nine *Panax* chloroplast genomes were compared with those of four model organisms. The number of codons with large frequency differences (ratio >= 2 or<= 0.5) was highest between *Panax* species and *Escherichia coli* (*24–25* codons), followed by *Arabidopsis thaliana* (12 codons for all species), *Saccharomyces cerevisiae* (*9–10* codons), and *Nicotiana tabacum* (*6–8* codons). Conversely, the number of codons with similar usage frequencies (ratio 0.5-2) was highest for *N. tabacum* (53–55 codons) and *S. cerevisiae* (51–52 codons), followed by *A. thaliana* (49 codons for all species) and *E. coli* (36–37 codons) (**Supplementary Figure S1**; **Supplementary Table 6**). These results indicate that *N. tabacum* had the closest codon-frequency similarity among the tested organisms, whereas *E. coli* showed the greatest divergence. Because yeast and plant plastids have fundamentally different expression systems, the apparent similarity with *S. cerevisiae* should be interpreted as codon-frequency similarity only and not as evidence of plastid expression compatibility.

## Discussion

### A/T-ending codon preference is a conserved feature of *Panax* chloroplast genomes

The present study systematically analyzed the codon usage patterns of chloroplast genes across nine *Panax* species. A consistent and prominent finding was the strong preference for codons ending in A or T across all species, as evidenced by GC3 values of 30.15%–30.52% and the observation that 96.6% of preferred codons (RSCU > 1) ended in A or T. This A/T-ending bias is a well-documented characteristic of chloroplast genomes in dicotyledonous plants and has been reported in numerous other plant lineages (Sueoka, 1999; Wu et al., 2025; Yan et al., 2025; Zhang et al., 2025; Zhu et al., 2026). The remarkable consistency of this pattern across all nine *Panax* species, despite their geographic and morphological diversity, suggests that A/T-ending codon preference is a deeply conserved feature of the *Panax* chloroplast genome, likely reflecting both the compositional constraints of the chloroplast genome and the evolutionary pressures acting on plastid gene expression.

### Multiple factors may shape CUB in *Panax* chloroplasts

The integrated analysis of ENC plots, PR2 plots, and neutrality plots suggests that *Panax* chloroplast CUB is shaped by multiple factors rather than by GC mutational pressure alone. In the neutrality plot, the slope-based estimates were low, but the GC12-GC3 correlations were weak and statistically non-significant. Consequently, these percentages should not be interpreted as exact measurements of the relative contributions of mutation and selection. Instead, the combined results support a cautious interpretation in which compositional constraints, strand-specific mutational bias, gene-level functional constraints, and possible weak selection jointly contribute to the conserved codon usage patterns observed in *Panax* chloroplast genes.

The ENC-plot analysis also supports a cautious interpretation. Quantitative classification relative to the expected ENC curve showed that most retained genes were above, rather than below, the expected curve: across all species, 103 genes (22.01%) were below the curve, 15 genes (3.21%) were on or near the curve, and 350 genes (74.79%) were above the curve (**Supplementary Table 9**). Thus, the ENC plot does not provide evidence that most genes have lower ENC values than expected from GC3s alone. The subset of genes below the curve may reflect factors beyond GC3s, including gene-level functional constraints or weak selection, whereas the large proportion of genes on/above the curve reinforces that ENC-plot results should not be used alone to infer a predominant selective mechanism. The PR2-plot analysis revealed asymmetric usage of complementary bases at the third codon position (T3 > A3 and G3 > C3 in most species), which may reflect natural selection, strand-specific mutational biases, or other compositional constraints. The relatively high mean ENC values (52.08-52.55) indicate that the overall degree of codon bias in *Panax* chloroplast genomes is moderate. The 11 shared candidate optimal codons were all A/T-ending, consistent with the overall nucleotide composition of the plastome. However, because no empirical expression or tRNA-abundance data were analyzed, we avoid presenting these codons as experimentally validated optimal codons or as direct evidence of translational selection.

### Highly conserved codon usage patterns reflect the close evolutionary relationships within *Panax*

A striking feature of the present analysis is the remarkable similarity in codon usage patterns across all nine *Panax* species. The GC content at all three codon positions, the number and identity of preferred codons, HF codons, and candidate optimal codons were nearly identical across species, with only minor quantitative differences. The 11 shared candidate optimal codons and 44 core HF codons were conserved across the genus. This high degree of conservation is consistent with the close phylogenetic relationships within *Panax* and suggests that the codon usage landscape of the *Panax* chloroplast genome has remained broadly stable throughout diversification of the genus. Similar conservation of codon usage patterns has also been reported in other plant groups, including Aconitum, Gnetales, Taraxacum, and Fagopyrum species (Yang et al., 2023; Yang et al., 2024a, b; Wei et al., 2026).

The COA further supports this pattern of conservation, with the first two principal axes collectively explaining only 18.28%-19.33% of the total variation and leaving approximately 80.67%-81.72% distributed across higher axes. This pattern indicates that codon usage variation is distributed across multiple axes. The significant correlations between Axis 1 and both GC3s and ENC suggest associations with base composition and codon-bias strength, but these associations should not be interpreted as proof that translational selection is the primary causal factor (Chen et al., 2026; Shen et al., 2024). We also summarized annotated tRNA repertoires from the nine plastomes (**Supplementary Table 8**), which showed 36–37 tRNA features and 29–30 unique tRNA genes per species. Mapping the 11 shared candidate optimal codons to annotated tRNA anticodons showed that nine codons had exact or wobble-compatible annotated tRNA support in all nine species, GGT had support in five species, and CCT had no direct annotated tRNA support under the simple pairing rules used here (**Supplementary Table 12**). This annotation-level summary provides useful context, but tRNA gene copy number is not equivalent to tRNA abundance or translational demand; transcriptomic or tRNA-expression data would be required to directly test codon-anticodon adaptation (Brule and Grayhack, 2017; Novoa et al., 2012; Wilusz, 2015).

### The suitable heterologous expression hosts for *Panax* chloroplast genes

The differences in codon usage patterns between *Panax* chloroplast genomes and the four model organisms likely reflect distinct translational systems and evolutionary constraints. The pronounced divergence from *Escherichia coli* is consistent with previous studies showing that codon usage can differ substantially between bacteria and plant organelles. *N. tabacum* showed the closest codon-frequency similarity among the tested organisms, which is consistent with its plant plastid background and its common use in chloroplast transformation studies. However, codon usage similarity alone cannot determine heterologous expression efficiency, which also depends on promoter and untranslated-region compatibility, RNA editing, mRNA stability, tRNA abundance, translation machinery, protein folding, and the transformation system (Gustafsson et al., 2004; Gorochowski et al., 2015; Novoa et al., 2012). The similarity observed with *S. cerevisiae* likely reflects general compositional features and should not be taken to imply suitability for plastid gene expression. We therefore describe *N. tabacum* as the most similar tested organism in codon usage rather than definitively the most suitable host.

## Conclusion

In conclusion, this study provides a comparative analysis of CUB in the chloroplast genomes of nine *Panax* species. The retained CDSs show a moderate and highly conserved codon usage pattern, characterized by a pronounced preference for codons ending in A or T. We identified 11 candidate optimal codons shared by all nine species, all of which terminate in A or T. ENC, PR2, neutrality-plot, and COA analyses suggest that Panax chloroplast codon usage cannot be explained by GC mutational pressure alone and is likely shaped by a combination of compositional constraints, strand-specific bias, gene-level constraints, and possible weak selection. However, because these analyses are indirect and no independent expression or tRNA-abundance data were included, mechanistic conclusions should be treated cautiously. Comparative codon-frequency analysis indicates that *N. tabacum* has the closest codon usage similarity among the tested organisms, while *E. coli* is the most divergent. These findings provide a useful descriptive reference for Panax plastid codon usage and for future studies that integrate expression, tRNA, and functional validation data.

## Data Availability

Publicly available datasets were analyzed in this study. This data can be found here: The complete chloroplast genome sequences of the nine Panax species analyzed during the current study were retrieved from the NCBI database (https://www.ncbi.nlm.nih.gov/). The specific GenBank accession numbers are as follows: Panax ginseng (NC_006290), Panax japonicus (NC_028703), Panax major (NC_053713), Panax pseudoginseng (MW145449), Panax quinquefolius (NC_027456), Panax stipuleanatus (NC_030598), Panax trifolius (NC_037994), Panax vietnamensis (MF377623), and Panax zingiberensis (NC_043954).
